# ∆Np63/p40 correlates with the location and phenotype of basal/mesenchymal cancer stem‐like cells in human ER^+^ and HER2^+^ breast cancers

**DOI:** 10.1002/cjp2.149

**Published:** 2019-12-06

**Authors:** Yajing Liu, Marta Nekulova, Rudolf Nenutil, Iva Horakova, M Virginia Appleyard, Karen Murray, Jitka Holcakova, Michaela Galoczova, Philip Quinlan, Lee B Jordan, Colin A Purdie, Borivoj Vojtesek, Alastair M Thompson, Philip J Coates

**Affiliations:** ^1^ NCRC, University of Michigan Ann Arbor MI USA; ^2^ Regional Centre for Applied Molecular Oncology Masaryk Memorial Cancer Institute Brno Czech Republic; ^3^ Dundee Cancer Centre University of Dundee, Ninewells Hospital and Medical School Dundee UK; ^4^ Advanced Data Analysis Centre University of Nottingham Nottingham UK; ^5^ Department of Pathology Ninewells Hospital and Medical School Dundee UK; ^6^ Division of Surgical Oncology Dan L Duncan Comprehensive Cancer Center, Baylor College of Medicine Houston TX USA

**Keywords:** p63, ΔNp63, p40, breast, cancer stem cells, oestrogen receptor, HER2, aldehyde dehydrogenase, CD44

## Abstract

ΔNp63, also known as p40, regulates stemness of normal mammary gland epithelium and provides stem cell characteristics in basal and HER2‐driven murine breast cancer models. Whilst ΔNp63/p40 is a characteristic feature of normal basal cells and basal‐type triple‐negative breast cancer, some receptor‐positive breast cancers express ΔNp63/p40 and its overexpression imparts cancer stem cell‐like properties in ER^+^ cell lines. However, the incidence of ER^+^ and HER2^+^ tumours that express ΔNp63/p40 is unclear and the phenotype of ΔNp63/p40^+^ cells in these tumours remains uncertain. Using immunohistochemistry with p63 isoform‐specific antibodies, we identified a ΔNp63/p40^+^ tumour cell subpopulation in 100 of 173 (58%) non‐triple negative breast cancers and the presence of this population associated with improved survival in patients with ER^−^/HER2^+^ tumours (*p* = 0.006). Furthermore, 41% of ER^+^/PR^+^ and/or HER2^+^ locally metastatic breast cancers expressed ΔNp63/p40, and these cells commonly accounted for <1% of the metastatic tumour cell population that localised to the tumour/stroma interface, exhibited an undifferentiated phenotype and were CD44^+^/ALDH^−^. *In vitro* studies revealed that MCF7 and T47D (ER^+^) and BT‐474 (HER2^+^) breast cancer cell lines similarly contained a small subpopulation of ΔNp63/p40^+^ cells that increased in mammospheres. *In vivo*, MCF7 xenografts contained ΔNp63/p40^+^ cells with a similar phenotype to primary ER^+^ cancers. Consistent with tumour samples, these cells also showed a distinct location at the tumour/stroma interface, suggesting a role for paracrine factors in the induction or maintenance of ΔNp63/p40. Thus, ΔNp63/p40 is commonly present in a small population of tumour cells with a distinct phenotype and location in ER^+^ and/or HER2^+^ human breast cancers.

## Introduction

The human *TP63* gene encodes two major variants that differ in their N‐terminal sequences, with TAp63 containing a p53‐like transactivation domain and ΔNp63 (also known as p40) lacking this domain. ΔNp63/p40 is an important stem cell regulator in the epidermis and glandular epithelial tissues and is crucial for normal development of these tissues 1, 2, 3. In normal adult breast, p63 is expressed exclusively in myoepithelium and initial studies in neoplasia led to the suggestion that p63 (predominantly ΔNp63/p40) is a marker of oestrogen receptor negative (ER^−^), basal, squamous and metaplastic breast carcinomas 4, 5, 6, 7. Nevertheless, the role of p63 in breast malignancy remains unclear.

Breast cancer cells exhibit substantial phenotypic heterogeneity, including subpopulations of cells with stem cell‐like properties, termed cancer stem cells (CSCs; also called cancer‐initiating cells). Recent studies have highlighted the plasticity and heterogeneity of breast CSCs, where different CSC types exist within and between tumours 8, 9, 10, 11, 12, 13 and each subtype has a different effect on survival 8, 11. In particular, two distinct breast CSC types have been reported and classed as luminal versus basal, or as epithelial versus mesenchymal, defined by reciprocal expression of MM1 and CD271, or of aldehyde dehydrogenase (ALDH) and CD44, respectively 9, 10. In addition to the presence of CD44 and lack of ALDH, mesenchymal‐like CSCs occupy a peri‐stromal location, whereas ALDH^+^/CD44^−^ epithelial‐like CSCs are located more centrally within the tumour 10. With regard to p63 in breast cancer, ΔNp63/p40 promotes or maintains stem cell activities in murine models of triple negative breast cancer (TNBC) and human epidermal growth factor receptor 2 (HER2)‐driven basal cancer 14, 15, 16. ΔNp63/p40 also induces CSC‐like properties when overexpressed in luminal breast cancer cell lines *in vitro* 17, 18 and has been associated with the CD271^+^ population of basal‐type CSCs in luminal breast cancers and cell lines 9. In clinical samples, high level p63 is characteristic for basal‐like TNBCs, where ΔNp63/p40 regulates EGFR, basal differentiation and adhesion, and TAp63 associates with androgen receptor, *PTEN* mutation and improved survival 19, 20, 21, 22, 23, 24. p63 is also present in some ER^+^ and/or HER2^+^ cancers, although at lower levels than in TNBCs 4, 5, 6 and ΔNp63/p40^+^ cells have been reported to be unrelated to or to associate with a basal‐like phenotype 9, 20. The incidence of ER^+^/HER2^+^ cancers that contain a ΔNp63/p40^+^ tumour cell subpopulation is also uncertain, partly due to the presence of ΔNp63/p40 in normal myoepithelium, which is a common component of surgically removed human breast cancers 4, 25, 26. In this study, we used clinical samples and cell line models to re‐investigate the incidence of p63 and its isoforms in human breast cancer. We also investigated the phenotype of these cells and their relationship with CSC subtypes.

## Materials and methods

### Human breast cancer samples

In compliance with the Declaration of Helsinki, permission for the use of anonymised excess human tissues was approved following local ethical committee review (the Tissue Access Committees at the Tayside Tissue Bank, Dundee, UK and the Biobank of clinical samples at the Masaryk Memorial Cancer Institute, Brno, Czech Republic). Tissue samples comprised axillary lymph node metastatic deposits of 33 consecutive ductal breast carcinomas diagnosed in Brno with metastasis greater than 2 mm in size (at least pN1) and tissue microarrays (TMAs) of unselected primary breast cancer tissues from patients in Dundee who had not received treatment before surgery. Tissues used for TMAs and patient characteristics including *TP53* mutation status and immunochemical characterisation following REMARK guidelines are provided in references 27, 28. In total, 212 tumours were analysed, comprising 39 triple‐negative cancers and 173 ER^+^ and/or HER2^+^ cancers.

### Cell culture and treatments

MCF7 (and derivative lines LCC1 and LCC9), T47D, CAMA‐1 and ZR‐75‐1 (ER^+^/HER2^−^); SK‐BR‐3 (HER2^+^) and BT‐474 (ER^+^/HER2^+^); TNBC cell lines BT‐20, BT‐549, HCC1806, MDA‐MB‐157, MDA‐MB‐231, MDA‐MB‐453, MDA‐MB‐468 and PMC‐42; and non‐transformed MCF10A cells (basal phenotype) and FaDu (squamous cell carcinoma) were obtained from ATCC, ECACC or DSMZ. Cells were grown at 37 °C with 5% CO_2_ in high glucose DMEM or McCoys 5A (for SKBR3), each with 10% fetal bovine serum (all from Invitrogen, Paisley, UK). MEBM with growth supplements (SingleQuots; Lonza, Slough, UK) was used for MCF10A. Cells were split regularly when approximately 80% confluent and all assays were performed when cells were subconfluent.

For mammosphere growth, cells were detached by trypsin and single suspensions prepared by passing through a 21G needle at least five times. Cells were counted and checked for doublets in a haemocytometer and 5000 cells/cm^2^ were plated in dishes pre‐coated with 1% poly(2‐hydroxy‐ethyl‐methacrylate) (Sigma‐Aldrich, Dorset, UK) in 90% ethanol. Cells were grown in standard mammosphere culture conditions; serum‐free DMEM/F12 (Invitrogen) containing B27 (Invitrogen) and SingleQuots growth factor supplements (Lonza) 11.

### Tumour xenografts

Formalin fixed paraffin embedded tissue blocks of MCF7 and MDA‐MB‐231 cells were available from previous xenograft studies 29. In brief, cells had been injected subcutaneously in female nu/nu immunocompromised mice (Harlan, Loughborough, UK). MCF7 xenografts were supplemented with slow release 17β‐estradiol pellets (0.72 mg per pellet; Innovative Research of America, FL, USA). Tumours were excised, fixed in 10% formalin overnight and processed into paraffin wax using the same procedures as human tissue samples. Animal procedures were carried out under project licences 60/3405 and 60/3729 according to the guidelines of the UKCCCR.

### Reverse transcription‐quantitative PCR

RNA was extracted from 5 × 10^6^ cultured cells and DNA removed by on‐column DNase digestion (RNeasy; Qiagen, West Sussex, UK). RNA integrity and concentrations were determined using an Agilent 2100 Bioanalyser (Agilent Technologies, Stockport, UK) before cDNA synthesis from 1 μg RNA using random primers and AffinityScript reverse transcriptase (Agilent). QuantiTect SYBR Green PCR with UNG treatment (Qiagen) was used for amplification and quantification in a Mx3005P QPCR System (Agilent) during 40 PCR cycles. Data were analysed using MxPro QPCR software (Agilent). Changes in mRNA levels were calculated using the 2^−ΔΔCt^ method with *GAPDH* as the reference gene using primers from Qiagen QuantiTect Primer Assay. Primers for *TP63* isoforms (see supplementary material, Table S1) were custom synthesised (Sigma‐Aldrich).

### Western blotting

Cells were lysed in 150 mm NaCl, 1% NP‐40, 50 mm Tris pH 8.0, 5 mm EDTA containing protease inhibitor cocktail (Sigma‐Aldrich) and protein concentrations measured by Bradford assay (Bio‐Rad Laboratories, Watford, UK). Cell lysates were separated by SDS‐PAGE, blotted onto nitrocellulose membranes and probed with antibodies to p63 using standard conditions. Antibodies to actin (AC‐40, Sigma) were used as loading controls. Detection employed secondary peroxidase‐labelled anti‐rabbit or anti‐mouse (Dako, Agilent) followed by enhanced chemiluminescence (Amersham ECL; Fisher Scientific, Loughborough, UK).

### Immunostaining and scoring

Cultured cells grown on sterilised glass slides were fixed in −20 °C acetone/methanol (1:1) for 10 min and stored at −80 °C. Alternatively, cells or mammospheres were collected by centrifugation, fixed in formalin overnight, resuspended in agarose and processed to paraffin wax. Cell phenotyping used immunohistochemistry with primary antibodies to p63, epithelial membrane antigen (EMA, also called MUC1), ER‐alpha, ALDH and CD44 (see supplementary material, Table S2). Immunostaining for p63 employed monoclonal antibodies that recognise all p63 isoforms (pan‐p63 antibody, clone 6.1), an isoform‐specific TAp63 monoclonal (clone 4.1), and a monoclonal antibody (clone ΔNp63‐1.1) or affinity‐purified polyclonal sera specific for ΔNp63/p40. The specificity of these antibodies for their respective protein isoforms and lack of cross‐reactivity with the related p53 and p73 proteins has been demonstrated previously 21, 22, 30, 31. Single‐labelling used diaminobenzidine (DAB) (Sigma‐Aldrich) as chromogen with haematoxylin counterstaining. For dual staining, nickel sulphate/DAB (blue/grey) was used to detect the first antigen and the second antigen was detected with DAB (brown). Sections were either counterstained with nuclear fast red or left unstained. For immunofluorescence, primary antibodies from different species were applied together and detected with secondary fluorochrome‐conjugated goat anti‐rabbit and goat anti‐mouse (Invitrogen). Samples were mounted with ProLong Gold (Invitrogen) or Vectashield (Vector Laboratories, Peterborough, UK) with DAPI (4′,6‐diamidino‐2‐phenylindole) counterstain. Controls comprised sections/cells exposed to either antibody singly but detected with both secondary reagents.

In the cohort of metastatic tumours, percentages of positive cells were estimated for ER, progesterone receptor (PR), Ki67 and p63, with CD44 and ALDH as markers of mesenchymal or epithelial CSCs, respectively 9, 10. In TMAs, ER and PR were scored using the QuickScore 32, where percentage of cells is graded from 1 to 6 and intensity from 0 to 3, with percentage score × intensity score recorded to give values ranging from 0 to 18. ER^+^ tumours are defined as having a QuickScore >3 27. For Ki67, only percentage was recorded (range 0–6). Samples were divided into categories of low, medium or high Ki67^+^ cells, using three different cut‐offs to define low (QuickScore 1 [0–4%], QuickScore 2 [0–19%] or QuickScore 3 [0–40%] and QuickScore >4 [60–100%]) to define high Ki67. In both sets of samples, HER2 immunostaining was scored on the clinically relevant scale of 0–3, with scores of 2 or 3 being subjected to *in situ* hybridisation to assess gene amplification.

### Statistical tests

Experimental data were obtained from three independent repeats. Quantitative immunostaining data were obtained by counting the percentages of positive cells in at least 10 high‐power microscope fields. Two‐tailed Student's *t*‐test was used for cell culture and Mann–Whitney *U* test for xenograft data due to the uneven and non‐random distribution of positive cells in tumour samples. Comparison of clinical data employed chi‐square, or Kaplan–Meier and Mantel–Cox log‐rank tests for survival analyses. *P* values <0.05 were considered significant.

## Results

### ∆Np63/p40^+^ cells are commonly present in luminal‐type human breast carcinomas

p63, specifically ∆Np63/p40, is present in the nuclei of normal basal/myoepithelial cells and pan‐p63 or ∆Np63/p40 antibodies are therefore used as a clinical marker to distinguish invasive from *in situ* lesions 4, 5, 6, 7, 25, 26. To investigate the incidence and phenotype of ∆Np63/p40^+^ tumour cells in human breast carcinomas, we initially examined regional (axillary) lymph node metastases, where contaminating myoepithelial cells will not be present, with pan‐p63, TAp63 and ΔNp63/p40 antibodies. Overall, 3 of 6 (50%) metastatic TNBCs and 11 of 27 (41%) receptor‐positive tumours showed p63^+^ nuclei in a subpopulation of tumour cells, including 7 of 22 ER^+^ and/or PR^+^ tumours and 5 of 6 HER2^+^ cancers (including the ER^+^/HER2^+^ cancer). Staining was seen using pan‐p63 and ∆Np63/p40 antibodies, but TAp63 was not seen in tumour cells (occasional lymphocytes were pan‐p63^+^ and TAp63^+^, as previously reported 24, 30). Less than 2% of the malignant cells were ∆Np63/p40^+^ in 8 of the 11 positive ER^+^ or HER2^+^ tumours, with 5 to 15% in the other three (see supplementary material, Table S3). Thus, using a standard cut‐off of 10% positive cells, only 2 of 33 (6%) of these tumours would be classified as ‘p63‐positive’.

**Figure 1:**
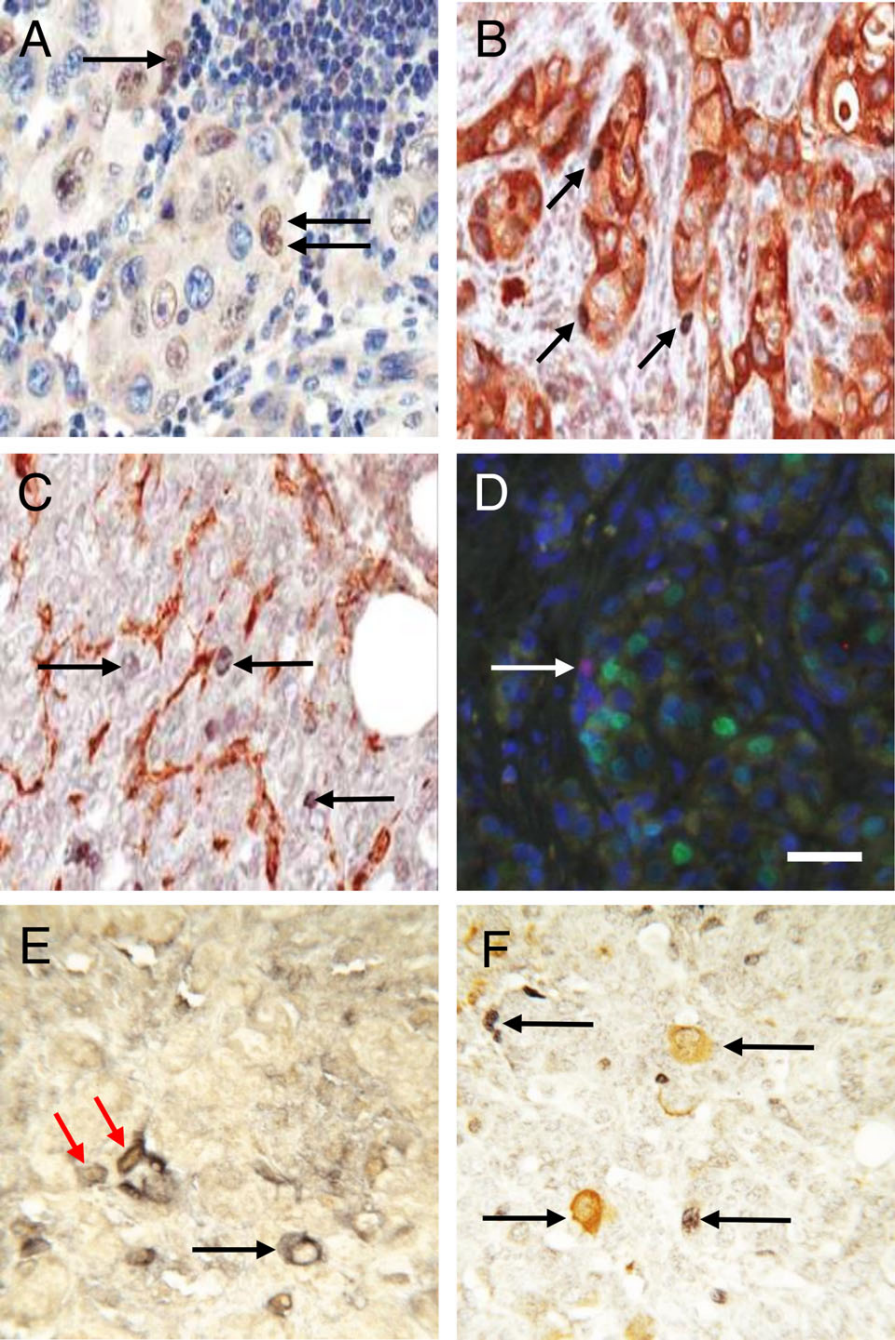
ΔNp63/p40 in human locally metastatic ER^+^ luminal breast cancer. (A) Immunohistochemistry for ΔNp63/p40 shows nuclear staining (brown) of a subpopulation of tumour cells in a local lymph node. Nuclei are counterstained with haematoxylin (blue). Three ΔNp63/p40^+^ cells are indicated by arrows. (B,C) Double‐labelling immunohistochemistry for ΔNp63/p40 (blue/grey) and (B) EMA (brown) or (C) SMA (brown). Arrows indicate ΔNp63/p40^+^ cells. (D) Immunofluorescence for ΔNp63/p40 (red) and ER (green) with DAPI counterstain (blue). The arrow indicates a ΔNp63/p40^+^ cell. (E) CD44 (blue/grey) and ΔNp63/p40 (brown). The red arrows indicate two adjacent cells that are CD44^+^ and ΔNp63/p40^+^, the black arrow indicates a CD44^+^ ΔNp63/p40^−^ cell. (F) ALDH (brown) and ΔNp63/p40 (blue/grey). Arrows indicate ΔNp63/p40^+^ or ALDH^+^ cells. Scale bar = 50 μm.

∆Np63/p40^+^ cells were preferentially located at the tumour/stroma interface in metastatic deposits (Figure 1A–F). Dual‐labelling indicated that ∆Np63/p40^+^ cells were not positive for smooth muscle actin (SMA), a reliable marker of cancer‐associated myoepithelial cells 25, and were EMA^−^ (a luminal epithelial differentiation marker) and ER^−/weak^ in ER^+^ cancers (Figure 1B–D). We also examined two markers of breast CSC subtypes, CD44 and ALDH. These markers varied from 0 to 90% of tumour cells in different tumours (see supplementary material, Table S3). ΔNp63/p40^+^ cells were never ALDH^+^, but a subset of CD44^+^ cells were ΔNp63/p40^+^ (Figure 1E,F). There were no associations between the presence of a ΔNp63/p40^+^ population and the number of nodes involved, tumour grade, extent of tubule formation, or proliferation measured by Ki67 or mitotic counts (see supplementary material, Table S3).

We subsequently used double‐staining of ∆Np63/p40 and SMA on TMAs of primary breast cancer tissues from patients who had not received therapy prior to surgery. Only ∆Np63/p40^+^ cells that were SMA^−^ were scored to exclude contaminating myoepithelial cells in these primary tumours. Of the 212 samples with sufficient tumour available for analysis, 173 were ER^+^ and/or HER2^+^, of which 100 (57.8%) contained at least one identifiable ΔNp63/p40^+^/SMA^−^ tumour cell. As with metastatic deposits, these cells were often seen at the edge of tumour cell islands.

**Figure 2:**
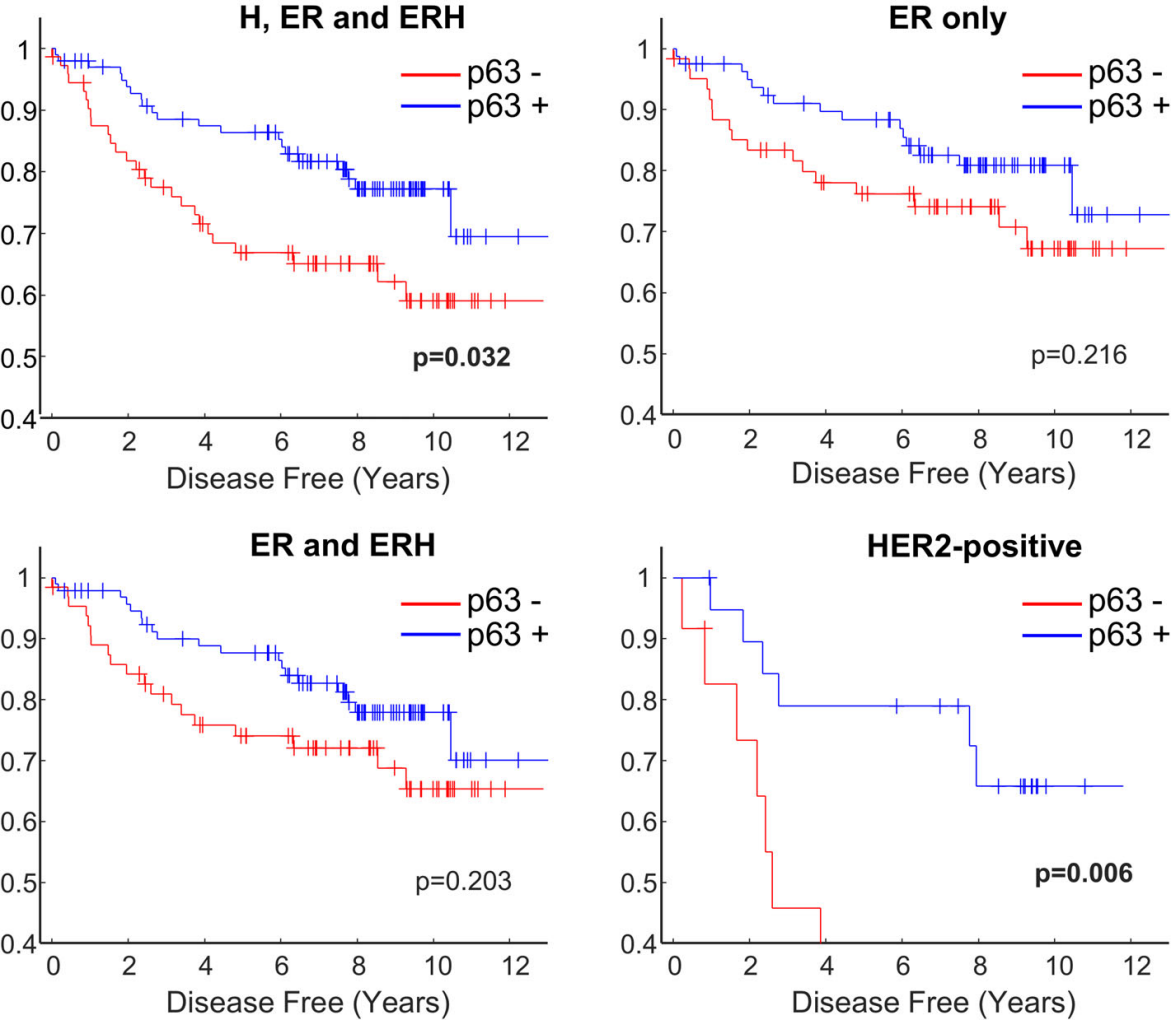
Kaplan–Meier plots of survival according to the presence or absence of ΔNp63/p40^+^ tumour cells in ER^+^/HER2^+^ breast cancers. Tumours were divided into those that contain a ΔNp63/p40^+^ population of SMA^−^ cancer cells (p63^+^; blue lines) and those in which this population was not seen (p63^−^; red lines) and correlated with disease‐specific survival. ER, ERH and H, tumours that are positive for ER or HER2, either singly or in combination (*n* = 173); ER only, tumours that are ER^+^ and HER2^−^ (*n* = 142); ER and ERH, tumours that are ER^+^/HER2^−^ or ER^+^/HER2^+^ (*n* = 158); HER2‐positive, HER2^+^ tumours that are ER^+^ or ER^−^ (*n* = 31). *P* values are Mantel–Cox log‐rank test.

Within the cohort of 173 receptor‐positive tumours, the presence of a p63/p40^+^/SMA^−^ cell population did not correlate with HER2 status, *TP53* mutation, tumour grade, lymph node status, proliferation using different Ki67 cut‐offs, or Nottingham prognostic index (NPI), but patients were more likely to be disease free at the time of last follow up (Table 1). Kaplan–Meier plots and log‐rank tests indicated that patients containing at least one ΔNp63/p40^+^/SMA^−^ tumour cell showed a more favourable outcome (*p* = 0.032). Sub‐grouping indicated that the effect on survival was confined to ER^−^/HER2^+^ cancers (*n* = 31; *p* = 0.006), with no statistically significant associations in patients with ER^+^/HER2^−^ or ER^+^/HER2^+^ tumours (Figure 2).

**Table 1:** Clinical, pathological and immunohistochemical data of 173 untreated primary ER^+^ and/or HER2^+^ breast carcinomas studied by TMA

	ΔNp63/p40^−^ (*n* = 73)	ΔNp63/p40^+^ (*n* = 100)	*P* value
ER	61	81	0.061
ERH	3	13	
H	9	6	
HER2	12	19	
*TP53* mutant	17	20	0.740
*TP53* wt	56	80	
Grades 1–2	45	59	0.729
Grade 3	26	40	
Positive nodes	36	43	0.527
Negative nodes	36	55	
Ki67 < 20%	20	35	0.411
Ki67 20–60%	47	55	
Ki67 > 60%	4	8	
With disease	26	21	0.0497
Disease free	47	79	
NPI ≤ 2.4	6	9	0.997
NPI 2.41–3.4	12	16	
NPI 3.41–5.4	35	50	
NPI > 5.4	17	23	

ΔNp63/p40^+^ cancers are defined as having at least one tumour cell that is ΔNp63/p40^+^ but SMA^−^. ER; positive for ER but not HER2. ERH; positive for both ER and HER2. H; positive for HER2 but negative for ER. HER2, positive for HER2 (=ERH + H); Values for Ki67 represent the percentage of tumour cells positive for Ki67 antigen; statistical significance was determined by chi‐square test. Data are shown for only one of the Ki67 cut‐offs; using <5% or <40% to define low Ki67 also indicated lack of statistical significance.

### ∆Np63/p40α is expressed in some luminal‐type breast cancer cell lines and increases in mammosphere cultures

To investigate whether any receptor‐positive breast cancer cell lines contain a similar population of ∆Np63/p40^+^ cells, we initially studied publicly available RNA‐Seq data of human cancer cell lines (https://www.ebi.ac.uk/arrayexpress E‐MTAB‐2706), containing data for 68 human breast cancer cell lines (see supplementary material, Figure S1). These analyses revealed that the highest *TP63* mRNA levels (comparable to a squamous carcinoma of the tongue cell line, FaDu) are seen in the basal cell lines HCC1806 and MCF10 series, although not all basal‐type lines show high levels (shown in green in supplementary material, Figure S1). *TP63* mRNA was also seen at low levels in some ER^+^ and HER2^+^ cell lines (see supplementary material, Figure S1). Similarly, we detected high levels of p63 in MCF10A (basal non‐malignant) and HCC1806 (basal carcinoma) cells by western blotting using a pan‐p63 and a ΔNp63/p40 specific antibody, at levels comparable to FaDu cells. All other cells tested were not positive by western blotting (see supplementary material, Figure S2A).

**Figure 3:**
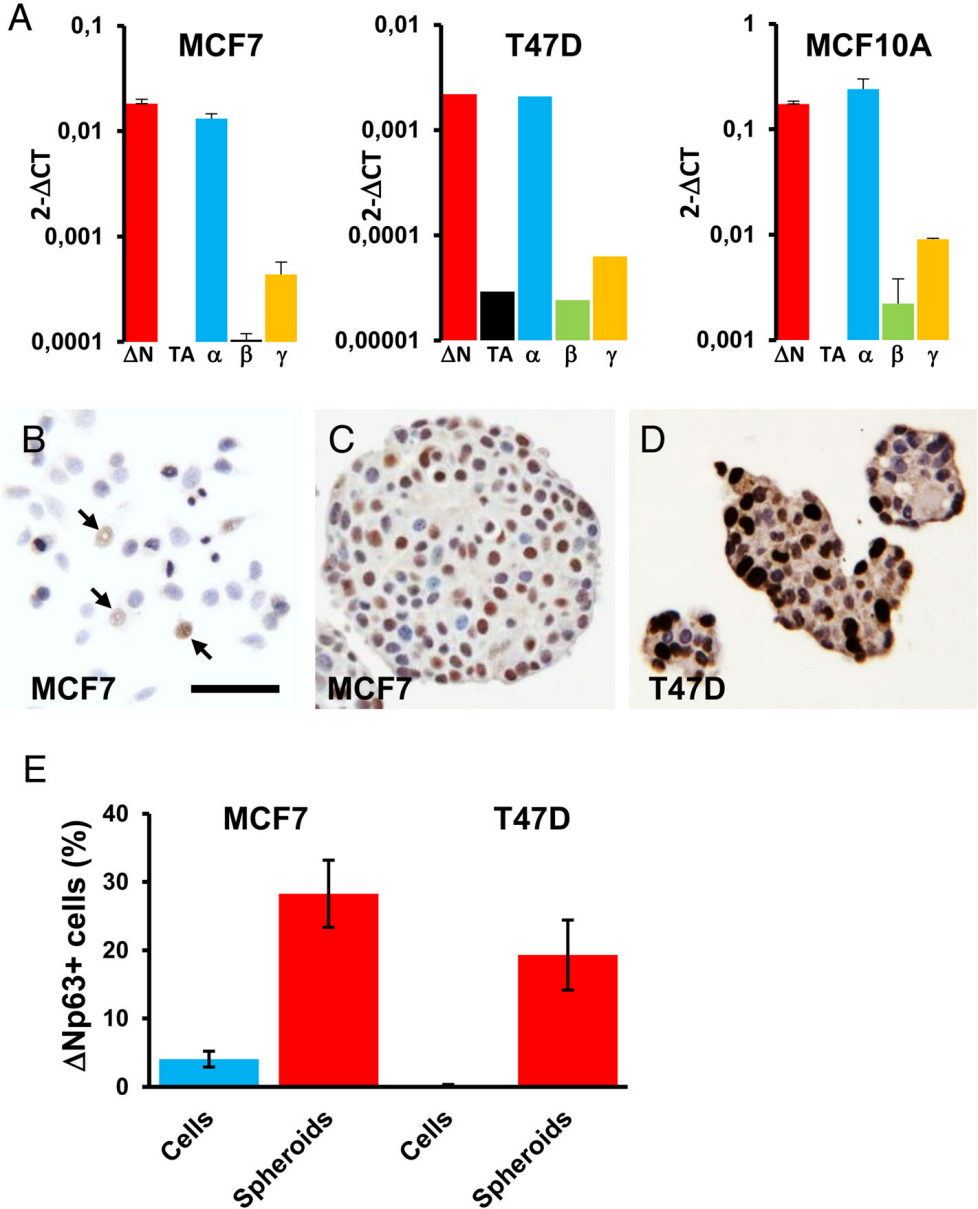
ΔNp63/p40 in luminal breast cancer cell lines. (A) RT‐qPCR data of the indicated *TP63* mRNA isoforms in MCF7, T47D and MCF10A cells. Values are given as relative levels normalised to *GAPDH*. Note that the *y* axes have different scales and are logarithmic to show the low levels of *TAp63*, *p63β* and *p63γ* mRNAs (error bars are SD of three independent experiments). (B–D) Representative images of immunohistochemical staining for ΔNp63/p40 in the indicated cells grown as monolayers under standard culture conditions (B) or as mammospheres (C,D). Arrows in (B) indicate ΔNp63/p40 positive cells. Scale bar = 50 μm for B–D. (E) Mean percentages of MCF7 and T47D cells that stain for ΔNp63/p40 grown as monolayer cells or as mammospheres (error bars are 95% confidence intervals of 30 spheroids from three independent experiments).

However, these data measure average mRNA or protein levels and do not discount the possibility that a small subpopulation of cells contain detectable levels of p63. We therefore used a combination of immunostaining and RT‐qPCR as a sensitive and specific method to detect *TP63* mRNA and to identify the specific mRNA isoforms. We identified variable levels of *TP63* mRNAs in two ER^+^ cell lines, MCF7 and T47D and in MCF10A cells (Figure 3A; note the different scales for the *y* axes). *ΔNp63* mRNA forms predominated over *TAp63* (*TAp63* was not detected in MCF7 or MCF10A and was 75‐fold lower than *ΔNp63* in T47D cells). For C‐terminal isoforms, *p63α* predominated in all three cell lines, with much lower levels of *p63β* and *p63γ* (Figure 3A). We then used immunocytochemistry to study p63 isoforms in cell lines. The majority of HCC1806 and MCF10A cells were positive using pan‐p63 and ∆Np63/p40 antibodies (see supplementary material, Figure S2B), in keeping with the western blotting and RNA‐Seq data. We also demonstrated the presence of pan‐p63 and ∆Np63/p40 in the nuclei of a small subpopulation of the ER^+^ cell lines, MCF7 (4%) and T47D (0.01%), and in the ER^−^/HER2^+^ BT‐474 cell line (0.1%) (Figures 3B and S2B). The ER^+^ MCF7 cell line derivatives, LCC1 and LCC9 also showed pan‐p63 and ∆Np63/p40 staining, similar to MCF7. The other cell lines tested did not contain pan‐p63^+^ or ΔNp63/p40^+^ cells (BT‐20, BT‐549, CAMA‐1, MDA‐MB‐157, MDA‐MB‐231, MDA‐MB‐453, MDA‐MB‐468, PMC‐42, SK‐BR‐3 and ZR‐75‐1). Staining was not seen using TAp63 specific antibody in any of the cell lines tested.

To assess whether ΔNp63/p40 is involved in stem cell phenotypes in receptor‐positive breast cancer cell lines, we also stained MCF7 and T47D cells grown as suspension spheroids (Figure 3C,D) (in our hands, BT‐474 cells do not form true mammospheres but exist in suspension as loose cell aggregates, similar to some other breast cancer cell lines 11). The percentages of ΔNp63/p40^+^ cells increased in both MCF7 and T47D cells when grown as mammospheres compared to monolayers (Figure 3E; *p* < 0.001 for each).

### ∆Np63/p40 marks a distinct subpopulation of MCF7 cells *in vivo*

**Figure 4:**
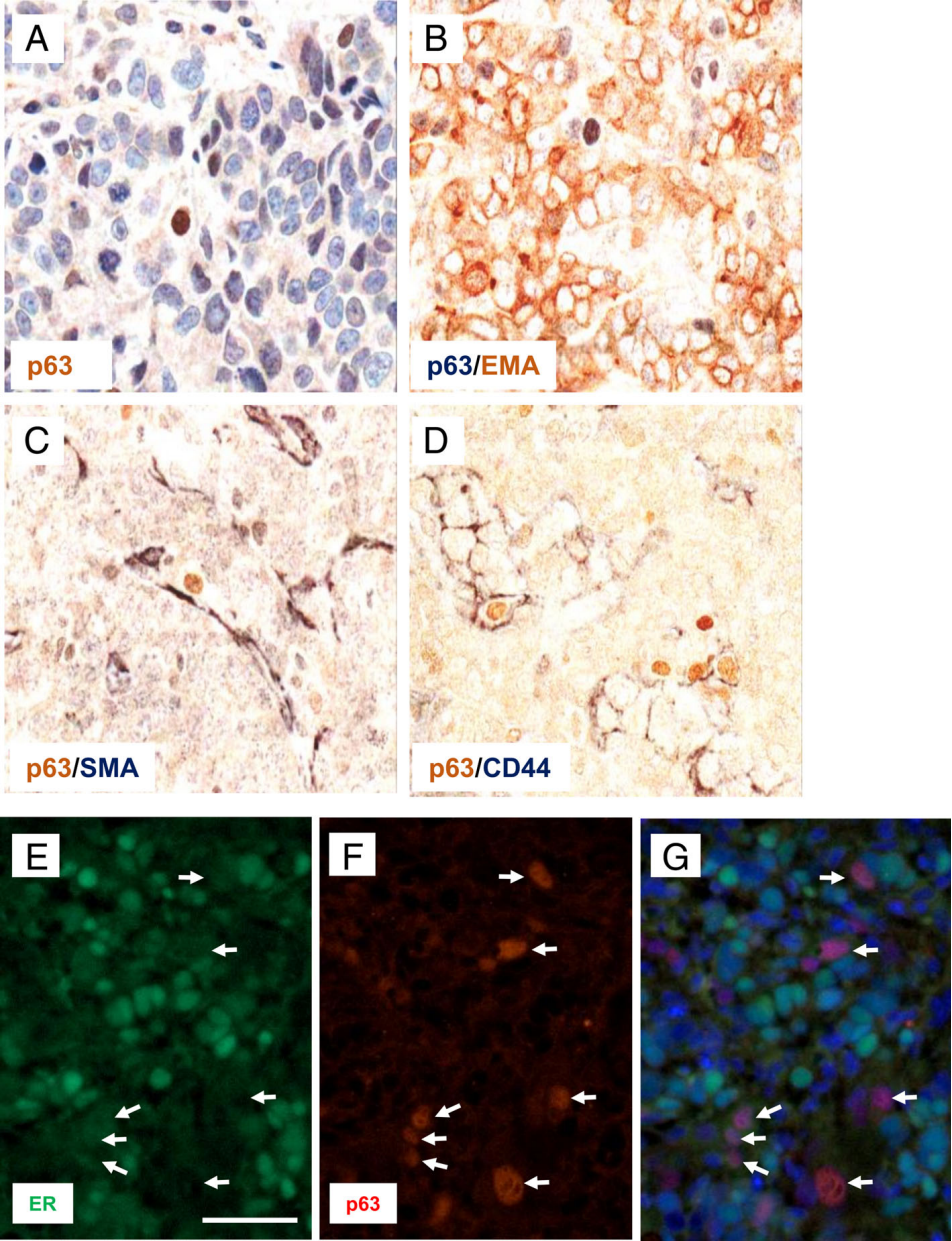
Localisation and phenotypic characterisation of ΔNp63/p40‐positive cells in MCF7 xenografts. Immunostaining of MCF7 cells grown as xenografts. (A) ΔNp63/p40 (p63) localisation to the nuclei of tumour cells adjacent to the tumour/stroma interface (ΔNp63/p40 in brown with haematoxylin counterstain). (B–D) Dual immunoperoxidase staining for (B) ΔNp63/p40 (p63, blue/grey) and EMA (brown); (C) ΔNp63/p40 (p63, brown) and SMA (blue/grey); (D) ΔNp63/p40 (p63, brown) and CD44 (blue/grey); no counterstain was used. (E–G) Immunofluorescence for ER in green (E) and ΔNp63/p40 (p63) in red (F). (G) The merged image with DAPI counterstain. Arrows indicate ΔNp63/p40^+^ cells. Scale bar = 50 μm.

Immunohistochemistry of MCF7 tumour xenografts demonstrated a small subpopulation of ∆Np63/p40^+^ cells throughout the tumour, often clustered together and close to the tumour/stroma interface (Figure 4A; see supplementary material, Figure S3 for additional examples of xenograft staining). MDA‐MB‐231 xenografts did not show any pan‐p63^+^ or ΔNp63/p40^+^ cells, in keeping with their absence in monolayer cultures. Similar to previous data from ER^+^ tumours, although the majority of MCF7 xenograft tumour cells were stained for the luminal marker EMA, less than 5% of the ∆Np63/p40^+^ cells were EMA^+^ (Figure 4B; see supplementary material, Figure S4B). ∆Np63/p40^+^ cells were often closely associated with SMA^+^ host stromal cells but were not themselves SMA^+^ and ∆Np63/p40 was present in a subpopulation (45.4 ± 8.2%) of CD44^+^ cells (Figure 4C,D; see supplementary material, Figure S3C,D). ∆Np63/p40^+^ cells were either negative or weakly stained for ER (Figure 4E–G). ALDH staining was not seen in MCF7 tumour xenografts, although this antibody identifies a subset of tumour cells and stromal cells in clinical material (see Figure 1).

## Discussion

Given the roles of ∆Np63/p40 in maintaining mammary stem/progenitor populations during normal development and in experimental breast cancers 14, 15, 16, 17, 18 and the occasional reports of pan‐p63 and ΔNp63/p40 in some non‐TNBCs 5, 9, 18, 20, 33, 34, 35, we re‐investigated p63 in human breast cancer. Pan‐p63 or ∆Np63/p40 antibodies are useful marker of normal myoepithelium to identify benign lesions and discriminate invasive from *in situ* cancers in clinical material 4, 26, which complicates the analysis of p63 in tumour cells of primary breast cancers. In particular, occasional pan‐p63 or ∆Np63/p40^+^ cells at the tumour periphery may represent myoepithelium rather than neoplastic cells. On the other hand, CSCs often exist at very low frequency 36, 37, 38 and may be localised in specific cell niches, including the tumour periphery 8, 9, 10, 13.

To avoid these problems, we initially examined locally metastatic tumours (which should not contain non‐neoplastic myoepithelium) and found that 41% of ER^+^, PR^+^ or HER2^+^ cancers contained pan‐p63^+^ and ∆Np63/p40^+^ cells, but not TAp63^+^ cells. The neoplastic nature of these cells was confirmed by their nuclear morphology. These cells were always a minor population (most commonly 1% or less) and were distributed non‐randomly within the metastatic tumours, predominantly at the tumour/stroma interface. We subsequently assessed TMAs of primary non‐TNBCs using double‐labelling for SMA and ∆Np63/p40 to avoid identifying contaminating basal/myoepithelial cells. More than 50% of these contained a ∆Np63/p40^+^ SMA^−^ subpopulation, sometimes represented by a single cell in one of the TMA cores. These data indicate that ∆Np63/p40^+^ tumour cells are common in receptor‐positive breast cancers, but are usually present at a very low frequency, unlike basal‐type TNBCs which often contain high numbers of ∆Np63/p40^+^ cells 7, 9, 19, 20, 24. We also found that two ER^+^/HER2^−^ lines (MCF7 and T47D) and BT‐474 cells (ER^−^/HER2^+^) show a small population of ∆Np63/p40^+^ cells, suggesting that these may be useful models to investigate the role of ΔNp63/p40 in these breast cancer subtypes.

In addition to determining incidence, we also investigated the phenotype of ΔNp63/p40^+^ cells in clinical samples and cell line models of ER^+^ and HER2^+^ breast cancer. Double‐labelling indicated that the ∆Np63/p40^+^ subpopulation lacked the luminal differentiation markers ER and EMA, in keeping with a relatively undifferentiated cell type within these tumours. In terms of stem cell markers, ΔNp63/p40 identified a subpopulation of CD44^+^ cells and were consistently negative for ALDH in patient samples and MCF7 xenografts. This pattern is similar to the ‘basal‐like’ breast CSC population identified by Kim *et al* based on expression of the basal marker CD271 and lack of the luminal differentiation marker MM1 9, and to the ‘mesenchymal‐like’ rather than ‘epithelial‐like’ CSCs of Liu and colleagues based on the CD44^+^/CD24^−^ phenotype and lack of ALDH 10. It is also noteworthy that ∆Np63/p40^+^ cancer cells localised at the periphery of tumour cell islands in primary and locally metastatic patient samples, the location of mesenchymal‐like CSCs 10, where stem cell activities require interactions with stromal cells and/or microenvironmental factors that constitute the CSC niche 13. That MCF7 xenografts showed a similar location provides additional evidence for stromal influences on the induction or maintenance of ∆Np63/p40 in these cells. Further evidence for a CSC‐like phenotype comes from the increase in ∆Np63/p40 in mammosphere growth conditions that enrich for CSC‐like cells in cell lines and primary tumours 39. These observations complement previous studies in which exogenous ∆Np63/p40 overexpression promoted a variety of CSC‐like properties in these cells 17, 18. Unfortunately, due to the nuclear location of ∆Np63/p40, we are unable to isolate live ∆Np63/p40^+^ cells to directly assess their mammosphere forming ability or other CSC properties.

Although ∆Np63/p40 has pro‐survival functions in mammary stem/progenitor cells 40 and in basal/HER2‐driven experimental breast cancers 15, 16, we found that a ΔNp63/p40^+^ cell population was not associated with poor patient survival in ER^+^ or HER2^+^ breast cancers, mirroring previous observations 18, 34. In contrast, patients with HER2^+^ cancers containing a ∆Np63/p40^+^ population had improved survival. Of note, these patient samples pre‐date the clinical use of trastuzumab. Other studies have shown positive and negative associations between ∆Np63/p40 and HER2 (or other ERBB family members) 18, 23, 40, 41, 42 and it is therefore likely that HER2‐∆Np63/p40 crosstalk accounts for our observations. Investigation of ∆Np63/p40 as a marker of improved prognosis deserves further study, potentially in the setting of HER2 directed clinical trials. We also note that our findings are relevant to daily diagnostic routine, where scattered pan‐p63^+^ or ΔNp63/p40^+^ cells are sometimes seen and may represent a challenge to report invasive breast cancer. In these rare doubtful cases of ‘scattered’ versus ‘rim‐like’ staining, the potential that these cells represent ΔNp63/p40^+^ tumour cells rather than myoepithelial cells should be considered and alternative markers (SMA, smooth muscle myosin heavy chain or calponin) would be useful, with the staining pattern being interpreted in the context of the morphology.

It is worth noting that studies such as ours that identify rare subpopulations of cells within a tumour mass are not possible using data from current array‐based or next‐generation sequencing technologies, including commercial prognostic breast cancer arrays, such as OncotypeDX, MammaPrint, PAM50/Prosigna or EndoPredict (reviewed in 43). These targeted or non‐targeted transcriptomic approaches measure average mRNA levels in tumour lysates and are unlikely to identify rare cellular populations due to lack of sensitivity within the overall transcriptome of the whole tumour tissue. Moreover, it is not possible to perform phenotypic analysis of individual cell populations from average transcript levels in a tissue lysate. Thus, whilst genomic, transcriptomic and proteomic data have been extremely useful for identifying broad differences in cancer sub‐types and have prognostic value 43, they hide single cell heterogeneity. As an important aside of relevance to p63, array based data generally do not identify alternative transcripts, so that the identification of *TP63* mRNA does not distinguish between TAp63 in lymphocytes, ΔNp63/p40 in myoepithelium or ΔNp63/p40 in tumour cells. Thus, at the present time, studies that identify and phenotype rare subpopulations of heterogeneous cells require *in situ* approaches such as immunohistochemistry. In the future, application of single cell transcriptomics will be valuable, although even here the analysis of rare cell populations would require data from thousands to millions of individual cells.

In conclusion, ΔNp63/p40 is expressed by a small subpopulation of cells in many ER^+^ and HER2^+^ human breast cancers. That metastatic deposits do not have a higher incidence of ∆Np63/p40^+^ cells argues against a specific role in driving metastasis, corroborated by the lack of correlation with clinical parameters including node status. The phenotype and location of ∆Np63/p40^+^ cells suggest that they represent basal/mesenchymal‐like rather than epithelial‐like CSCs, identifying ∆Np63/p40 as an additional marker of the phenotypic heterogeneity of breast cancer cell populations 44. Given the previous evidence that exogenous ΔNp63/p40 induces stem/progenitor phenotypes in mammary epithelial cells, including luminal‐type cancer cells *in vitro* 17, 18, our demonstration of endogenous ∆Np63/p40 in luminal cancers requires further study to help understand breast cancer stem/progenitor cell populations and provide additional avenues for therapeutic intervention.

## Author contributions statement

YL and MN performed experimental work and analysed data. RN and IH identified and analysed metastatic samples. MVA and KM performed xenografts. JH and MG performed experimental work. PQ collated data for TMAs and contributed to statistical analysis. CAP and LJB identified and characterised TMA samples. BV provided antibody resources and was involved in study design and supervision. AMT and PJC conceived and designed the study. PJC wrote the draft manuscript. PJC, YL, MN, BV and AMT revised the manuscript. All authors contributed to manuscript revision and approved the final version.

## Supporting information


**Figure S1.** RNA Seq data for *TP63* of all available transformed and non‐transformed human breast cell lines
**Figure S2.** Immunochemical detection of ΔNp63/p40 in breast cell lines
**Figure S3.** Additional examples of immunostaining of MCF7 xenografts for ΔNp63/p40 (p63)

**Table S1.** Primer sequences used for RT‐qPCR
**Table S2.** Primary antibodies used for immunohistochemistry
**Table S3.** Pathology data and ΔNp63/p40 immunostaining of 33 locally metastatic breast carcinomas
