# Supplementary material for: ∆Np63/p40 correlates with the location and phenotype of basal/mesenchymal cancer stem‐like cells in human ER+ and HER2+ breast cancers
Source: J Pathol Clin Res. 2019 Dec 6;6(1):83–93. doi: 10.1002/cjp2.149 (PMC6966710; doi:10.1002/cjp2.149)
Supplement: Supplementary file 1 — Figure S1. RNA Seq data for TP63 of all available transformed and non‐transformed human breast cell lines Figure S2. Immunochemical detection of ΔNp63/p40 in breast cell lines Figure S3. Additional examples of immunostaining of MCF7 xenografts for ΔNp63/p40 (p63) [file CJP2-6-83-s001.docx]

**∆Np63/p40 correlates with the location and phenotype of basal/mesenchymal cancer stem-like cells in human ER^+^ and HER2^+^ breast cancers**

Liu Y *et al*. *J Pathol Clin Res* DOI: 10.1002/cjp2.149

**Supplementary Figures**

**
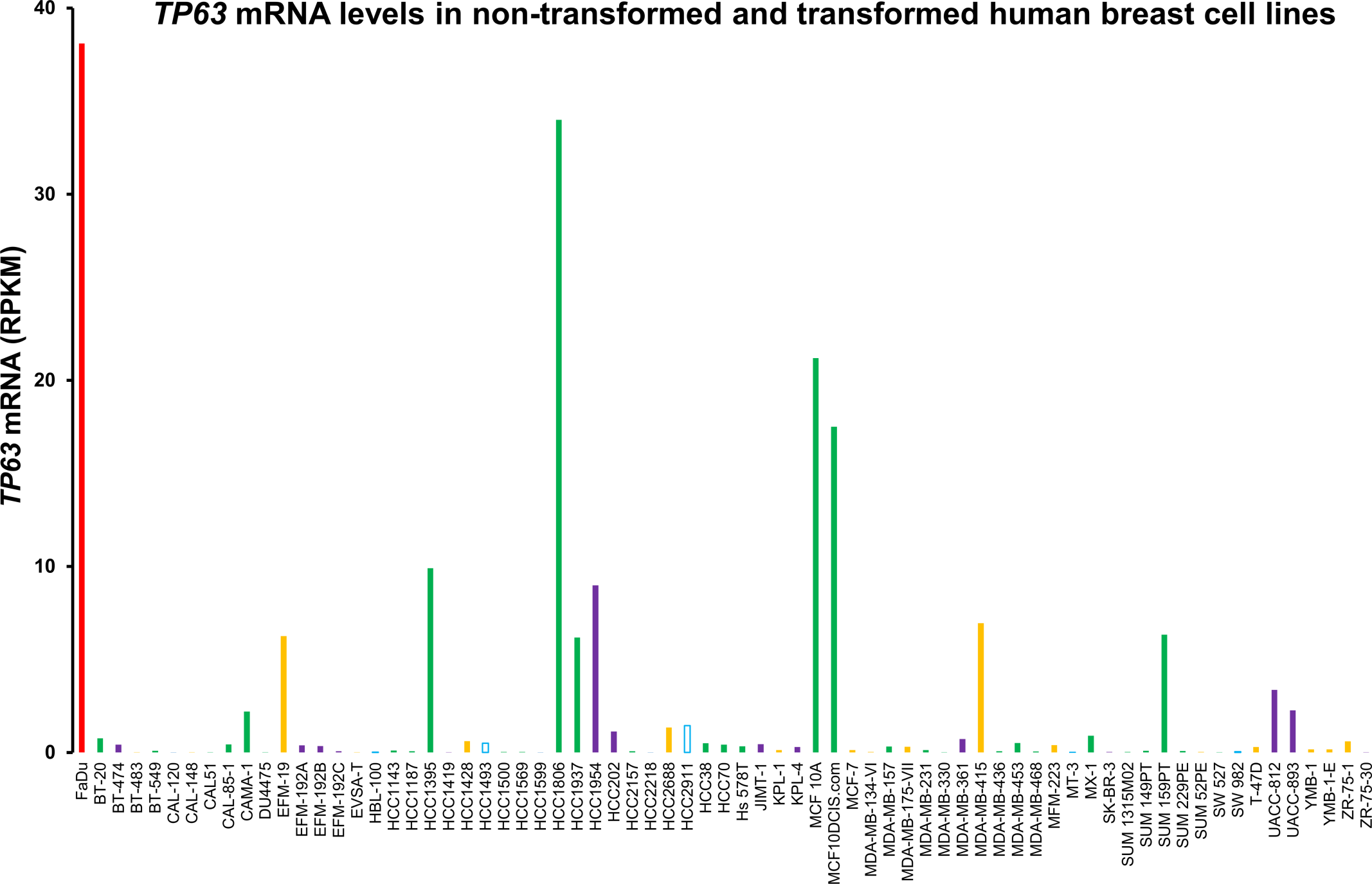
**

**Figure S1** *RNA Seq data for TP63 of all available transformed and non-transformed human breast cell lines (n=68).*

Values are RPKM (reads per kilobase of transcript per million mapped reads), obtained from https://www.ebi.ac.uk/arrayexpress/experiments/E-MTAB-2706/files). FaDu cells (squamous cell carcinoma of hyopharynx) are shown in red in the first column for comparison. Breast cell lines are arranged in alphabetical order. Green columns represent basal or triple-negative phenotype cells; orange columns represent luminal cell lines; purple columns represent HER2-amplified; empty columns represent cell lines of unclear phenotype.

**
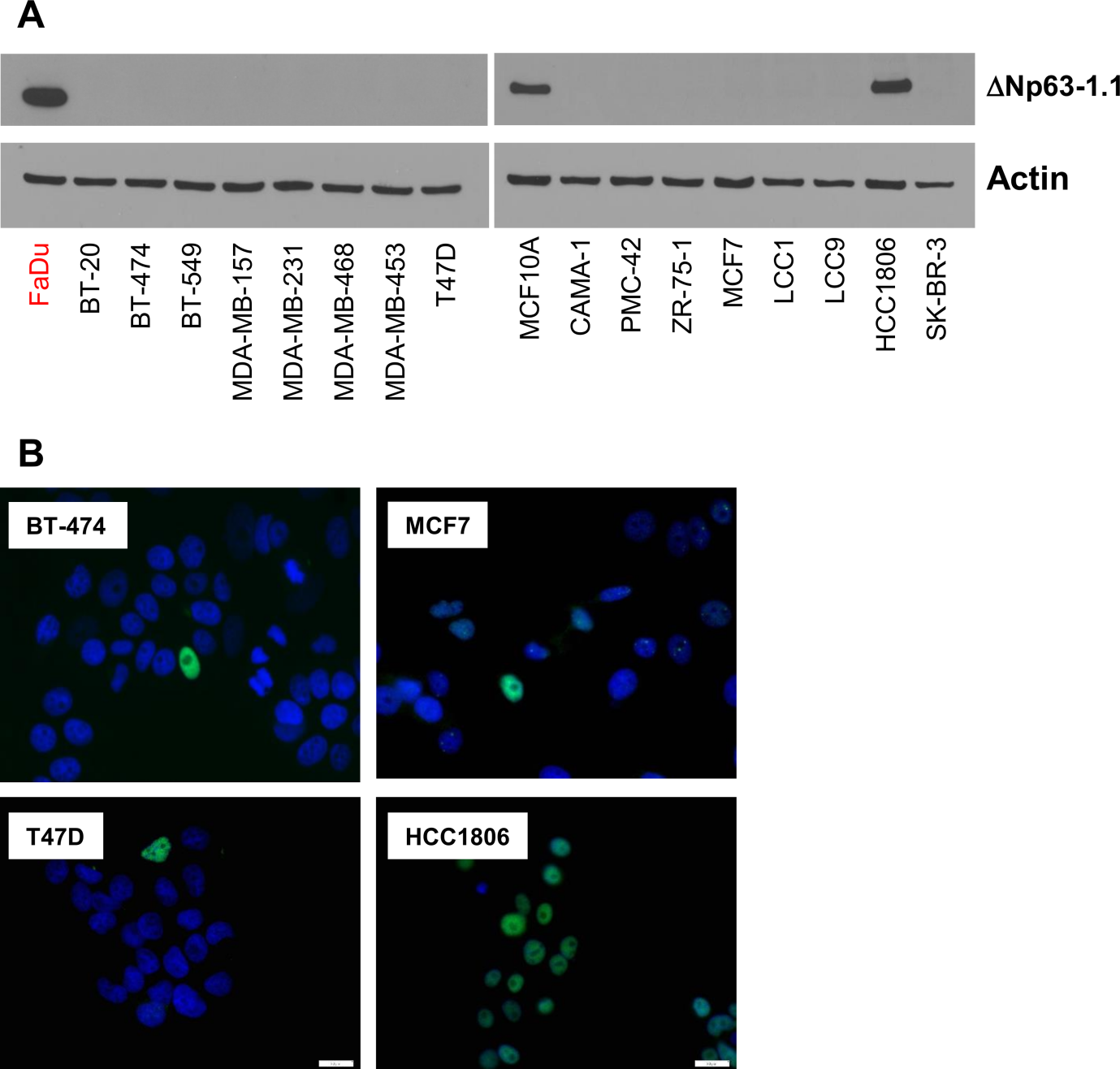
**

**Figure S2.** *Immunochemical detection of ΔNp63/p40 in breast cell lines.*

(A) Western blot of the indicated breast cell lines using ΔNp63-1.1 monoclonal antibody. FaDu cells are shown in the first lane as a control. Relative protein loading is shown with actin. (B) Examples of immunofluorescence staining of ΔNp63/p40 in subconfluent BT-474, MCF7, T47D and HCC1806 cells growing as monolayers. Positive cells are shown in green with nuclear DAPI counterstain (blue). Note that ΔNp63/p40^+^ cells are extremely rare in BT-474 and T47D cells and the examples show individual positive cells rather than representing the relative numbers of cells in each cell line. Scale bar = 20 μm.

**
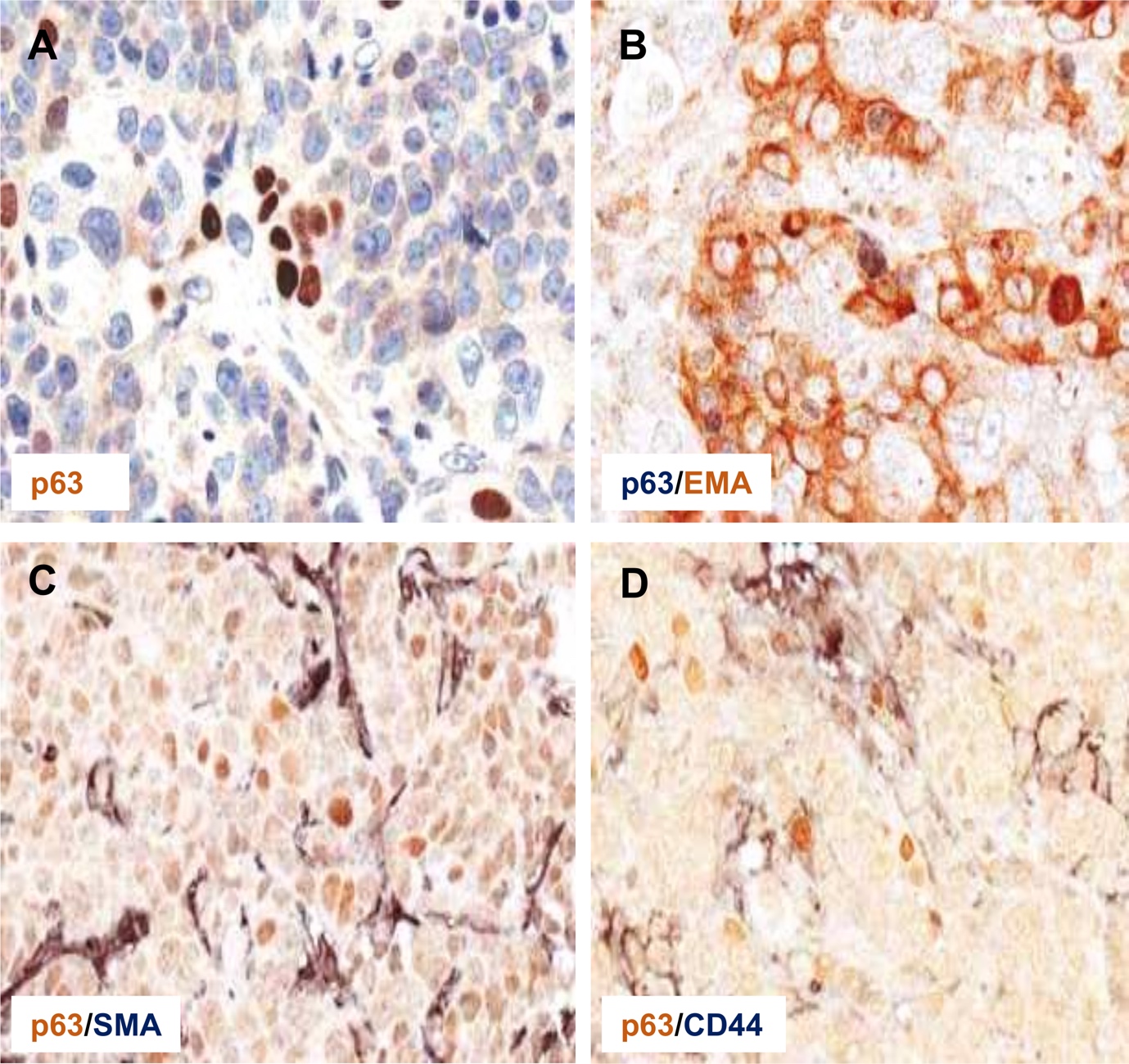
**

**Figure S3.** *Additional examples of immunostaining of MCF7 xenografts for ΔNp63/p40 (p63).*

(A) An area of xenograft tissue showing a higher percentage of ΔNp63/p40^+^ cells than shown in Figure 4, including an example of clustered positive cells. (B-D) Additional examples to those shown in Figure 4 of double-labelling for ΔNp63/p40 (p63) with the indicated antigens.
