# Supplementary material for: ∆Np63/p40 correlates with the location and phenotype of basal/mesenchymal cancer stem‐like cells in human ER+ and HER2+ breast cancers
Source: J Pathol Clin Res. 2019 Dec 6;6(1):83–93. doi: 10.1002/cjp2.149 (PMC6966710; doi:10.1002/cjp2.149)
Supplement: Supplementary file 2 — Table S1. Primer sequences used for RT‐qPCR Table S2. Primary antibodies used for immunohistochemistry Table S3. Pathology data and ΔNp63/p40 immunostaining of 33 locally metastatic breast carcinomas [file CJP2-6-83-s002.docx]

**∆Np63/p40 correlates with the location and phenotype of basal/mesenchymal cancer stem-like cells in human ER^+^ and HER2^+^ breast cancers**

Liu Y *et al*. *J Pathol Clin Res* DOI: 10.1002/cjp2.149

**Supplementary Tables**

**Table S1.** Primer sequences for *TP63* isoform mRNAs. Sequences are given 5’ 🡪 3’

| **Isoform** | **Forward primer** | **Reverse primer** |
| --- | --- | --- |
| *ΔNp63* | AGCCAGAAGAAAGGACAGCA | TCACTAAATTGAGTCTGGGCAT |
| *TAp63* | GTCCCAGAGCACACAGACAA | TAGCATGGACTGTATCCGCA |
| *p63α* | GAGGTTGGGCTGTTCATCAT | GAGGAGAATTCGTGGAGCTG |
| *p63β* | AACGCCCTCACTCCTACAAC | GCCAGATCCTGACAATGCTG |
| *p63γ* | GAAACGTACAGGCAACAGCA | GGGTACACTGATCGGTTTGG |

**Table S2.** Antibodies used for immunohistochemistry

| **Antigen** | **Supplier^1^, clone/Cat N^o^/citation** | **Species** | **Dilution** |
| --- | --- | --- | --- |
|  |  |  |  |
| ΔNp63 | ΔNp63-1.1 (p40) [21,22]  Gift of K Nylander, Umea [30]  ΔNp63-47 [31] | Mouse  Rabbit  Rabbit | 0.2μg/ml  1/20000  1/5000 |
|  |  |  |  |
| p63 | Sigma Aldrich, 4A4  In-house (PANp63-6.1) [31] | Mouse  Mouse | 1/1000  1/50 |
|  |  |  |  |
| TAp63 | In-house (TAp63-4.1) [31] | Mouse | 1/40 |
|  |  |  |  |
| ALDH1 | Becton Dickinson, 44/ALDH | Mouse | 1/2000 |
|  |  |  |  |
| CD44 | Leica; Novocastra, NVL-CD44 | Mouse | 1/4000 |
|  |  |  |  |
| EMA | Agilent; Dako E29 | Mouse | 1/5000 |
|  |  |  |  |
| SMA | Agilent; Dako 1A4 | Mouse | 1/1500 |
|  |  |  |  |
| ERα | Invitrogen; LabVision SP1 | Rabbit | 1/400 |
|  |  |  |  |
| PR | Invitrogen; LabVision SP2 | Rabbit | 1/1600 |
|  |  |  |  |
| HER2 | Leica; Novocastra CB11 | Mouse | 1/1000 |

All antibodies were used after antigen retrieval using 10mM citrate, pH 6.0 boiling for 15 minutes. Antibodies were applied at the indicated dilutions overnight at 4^o^C.

^1^Supplier address: Sigma-Aldrich, Dorset, UK; Becton Dickinson, Berkshire UK; Leica Biosystems, Milton Keynes, UK; Agilent, Stockport, UK; Invitrogen, Paisley, UK.

Citation numbers refer to the main article:

21. Orzol P, Nekulova M, Holcakova J, *et al.* ΔNp63 regulates cell proliferation, differentiation, adhesion, and migration in the BL2 subtype of basal-like breast cancer. *Tumour Biol* 2016; **37**: 10133-10140.

22. Nekulova M, Holcakova J, Gu X, *et al.* ΔNp63α expression induces loss of cell adhesion in triple-negative breast cancer cells. *BMC Cancer* 2016; **16**: 782.

30. Nylander K, Vojtesek B, Nenutil R, *et al.* Differential expression of p63 isoforms in normal tissues and neoplastic cells. *J Pathol* 2002; **198**: 417-427.

31. Nekulova M, Holcakova J, Nenutil R, *et al.* Characterization of specific p63 and p63-N-terminal isoform antibodies and their application for immunohistochemistry. *Virchows Arch* 2013; **463**: 415-425.

**Table S3**. Details of ΔNp63/p40 immunohistochemistry in lymph nodes of 33 metastatic breast cancers

| **Type** | **Subtype^1^** | **ΔNp63/p40** | **ER (%)** | **PR (%)** | **Ki67 (%)** | **HER2 IHC** | **HER2 FISH^2^** | **CD44^3^**  **(%)** | **ALDH1^3^**  **(%)** | **pT** | **T (mm)** | **pN** | **N pos^4^** | **N total^4^** | **Grade** | **Tubularity^5^** | **Mitoses/**  **2.5mm2** |
| --- | --- | --- | --- | --- | --- | --- | --- | --- | --- | --- | --- | --- | --- | --- | --- | --- | --- |
| Ductal | ER |  | 97 | 82 | 40 | 2 | No | nd | nd | 1c | 15 | 1a | 1 | 13 | 3 | 3 | 14 |
| Ductal | ER | 1-2% | 97 | 95 | 33 | 0 |  | 0 | 0 | 1c | 14 | 1a | 2 | 12 | 2 | 3 | 16 |
| Ductal | ER | 1 cell | 87 | 98 | 21 | 0 |  | 50 | 0 | 1c | 11 | 1a | 3 | 21 | 2 | 3 | 4 |
| Ductal | ER | 2% | 86 | 0 | 51 | 1 |  | <1 | 0 | 2 | 22 | 1a | 1 | 11 | 3 | 3 | 20 |
| Ductal | ERH | 15% | 42 | 43 | 26 | 3 | Yes | 0 | 0 | 2 | 25 | 2a | 6 | 15 | 3 | 3 | 13 |
| Ductal | ER |  | 98 | 67 | 16 | 0 |  | nd | nd | 2 | 45 | 1a | 1 | 15 | 3 | 3 | 15 |
| Ductal | ER | <1% | 98 | 0 | 29 | 0 |  | <0.1 | 0 | 2 | 32 | 1a | 1 | 22 | 3 | 3 | 12 |
| Ductal | ER | <1% | 84 | 0 | 26 | 0 |  | 60 | 0 | 2 | 38 | 2a | 4 | 9 | 3 | 2 | 35 |
| Ductal | ER |  | 93 | 91 | 27 | 0 |  | nd | nd | 1c | 19 | 2a | 4 | 9 | 2 | 2 | 25 |
| Ductal | ER |  | 95 | 0 | 14 | 1 |  | nd | nd | 4b | 20 | 1a | 1 | 12 | 2 | 2 | 100 |
| Ductal | ER |  | 99 | 100 | 53 | 0 |  | nd | nd | 2 | 28 | 1a | 2 | 10 | 2 | 2 | 57 |
| Ductal | ER | 5% | 90 | 21 | 48 | 1 |  | 0 | 80 | 2 | 50 | 1a | 1 | 16 | 3 | 3 | 27 |
| Ductal | ER |  | 88 | 1 | 24 | 3 |  | nd | nd | 1c | 17 | 1a | 2 | 14 | 3 | 3 | 10 |
| Ductal | ER |  | 78 | 45 | 14 | 1 |  | nd | nd | 1c | 12 | 2a | 4 | 9 | 2 | 2 | 25 |
| Ductal | ER |  | 87 | 67 | 13 | 0 |  | nd | nd | 2 | 26 | 1a | 1 | 13 | 2 | 2 | 20 |
| Ductal | H | 1% | 0 | 0 | 31 | 3 | Yes | 50 | 0 | 3 | 65 | 2a | 4 | 18 | 3 | 3 | 35 |
| Ductal | H | 1% | 0 | 0 | 22 | 3 | Yes | 0.1 | 0.1 | 1b | 7 | 1a | 1 | 10 | 3 | 3 | 17 |
| Ductal | ER |  | 97 | 51 | 20 | 1 |  | nd | nd | 1c | 18 | 1a | 1 | 18 | 2 | 3 | 17 |
| Ductal | ER |  | 93 | 100 | 20 | 0 |  | nd | nd | 2 | 35 | 2a | 5 | 13 | 2 | 2 | 34 |
| Ductal | ER |  | 92 | 0 | 36 | 1 |  | nd | nd | 1c | 20 | 2a | 6 | 17 | 3 | 3 | 16 |
| Ductal | H |  | 5 | 0 | 16 | 2 | Yes | nd | nd | 1c | 15 | 1a | 2 | 8 | 2 | 3 | 15 |
| Ductal | ER |  | 0 | 0 | 46 | 1 |  | nd | nd | 1c | 19 | 1a | 1 | 13 | 3 | 3 | 60 |
| Ductal | TN |  | 92 | 10 | 39 | 0 |  | nd | nd | 1c | 15 | 1a | 3 | 8 | 3 | 3 | 35 |
| Ductal | TN | 1% | 0 | 0 | 32 | 0 |  | 0.1 | 0 | 2 | 31 | 1a | 3 | 7 | 3 | 2 | 30 |
| Ductal | H | 1% | 0 | 20 | 58 | 3 | Yes | 10 | 90 | 2 | 30 | 2a | 7 | 14 | 3 | 2 | 28 |
| Ductal | TN |  | 78 | 90 | 26 | 0 |  | nd | nd | 1c | 14 | 1a | 1 | 13 | 2 | 3 | 7 |
| Ductal | H | 10% | 0 | 0 | 74 | 3 | Yes | 0.5 | 40 | 2 | 22 | 2a | 7 | 18 | 3 | 3 | 30 |
| Ductal | ER |  | 99 | 32 | 5 | 0 |  | nd | nd | 1c | 20 | 1a | 1 | 9 | 2 | 2 | 5 |
| Ductal | TN |  | 0 | 0 | 100 | 0 |  | nd | nd | 4b | 170 | 2a | 8 | 14 | 3 | 3 | 75 |
| Ductal | TN | <1% | 0 | 0 | 33 | 1 |  | 0 | 50 | 1b | 9 | 1a | 1 | 16 | 3 | 3 | 34 |
| Ductal | TN | 1% | 0 | 0 | 80 | 1 |  | 80 | 0 | 2 | 23 | 1a | 1 | 22 | 3 | 3 | 55 |
| Ductal | ER |  | 54 | 0 | 9 | 2 | No | nd | nd | 4b | 25 | 1a | 2 | 13 | 2 | 3 | 2 |
| Ductal | ER |  | 96 | 100 | 5 | 1 |  | nd | nd | 2 | 22 | 1a | 2 | 8 | 2 | 3 | 2 |

^1^ Subtype; ER = ER^+^ and HER2^-^; ERH = ER^+^ and HER2 amplified; H = ER^-^ and HER2^+^; TN = Triple negative

^2^ HER2 FISH; No = not amplified; Yes = amplified

^3^ CD44 and ALDH1 staining was performed only on samples with ΔNp63/p40^+^ cells. Percentages are the percentage of total tumour cells positive. nd = not done

^4^ N pos = number of lymph nodes with evidence of metastatic breast cancer; N total = total number of lymph nodes examined

^5^ Tubularity score 1: >75% of the tumour shows tubular structures with lumina; 2: 10-75% tubular pattern; 3 <10% tubule formation
